# Pilus Biogenesis in *Lactococcus lactis*: Molecular Characterization and Role in Aggregation and Biofilm Formation

**DOI:** 10.1371/journal.pone.0050989

**Published:** 2012-12-06

**Authors:** Virginie Oxaran, Florence Ledue-Clier, Yakhya Dieye, Jean-Marie Herry, Christine Péchoux, Thierry Meylheuc, Romain Briandet, Vincent Juillard, Jean-Christophe Piard

**Affiliations:** 1 INRA, UMR1319 Micalis, Domaine de Vilvert, Jouy-en-Josas, France; 2 Agro ParisTech, UMR 1319 Micalis, Jouy-en-Josas, France; 3 INRA, UR1196 GPL, Domaine de Vilvert, Jouy-en-Josas, France; University of Kansas Medical Center, United States of America

## Abstract

The genome of *Lactococcus lactis* strain IL1403 harbors a putative pilus biogenesis cluster consisting of a sortase C gene flanked by 3 LPxTG protein encoding genes (*yhgD*, *yhgE*, and *yhhB*), called here *pil*. However, pili were not detected under standard growth conditions. Over-expression of the *pil* operon resulted in production and display of pili on the surface of lactococci. Functional analysis of the pilus biogenesis machinery indicated that the pilus shaft is formed by oligomers of the YhgE pilin, that the pilus cap is formed by the YhgD pilin and that YhhB is the basal pilin allowing the tethering of the pilus fibers to the cell wall. Oligomerization of pilin subunits was catalyzed by sortase C while anchoring of pili to the cell wall was mediated by sortase A. Piliated *L. lactis* cells exhibited an auto-aggregation phenotype in liquid cultures, which was attributed to the polymerization of major pilin, YhgE. The piliated lactococci formed thicker, more aerial biofilms compared to those produced by non-piliated bacteria. This phenotype was attributed to oligomers of YhgE. This study provides the first dissection of the pilus biogenesis machinery in a non-pathogenic Gram-positive bacterium. Analysis of natural lactococci isolates from clinical and vegetal environments showed pili production under standard growth conditions. The identification of functional pili in lactococci suggests that the changes they promote in aggregation and biofilm formation may be important for the natural lifestyle as well as for applications in which these bacteria are used.

## Introduction

*Lactococcus lactis* belongs to the group of Lactic Acid Bacteria (LAB), which typically live in nutrient-rich ecological niches such as plants, gut mucus and milk. *L. lactis* is the most widely used species in dairy fermentation and is also the best characterized LAB and the first whose genome has been sequenced [1]. Numerous studies on the biology of this bacterium have opened doors to novel biotechnological applications in which lactococci are used both as cell factory and as delivery vehicles of beneficial molecules. These are antigens or cytokines for development of live mucosal vaccines or immune modulatory therapeutics [2], [3], [4], [5], [6], [7], enzymes or vitamins for improved health status of consumers [8], antimicrobials for improved food safety [9], and phage lysins and eventually holins to target food pathogens [10], [11] or to accelerate cheese ripening [12]. In those ongoing applications, interactions of the surface of lactococcal vehicles with the physical environment are likely to influence the behavior of bacteria and thereby their activity [13].

The surface of Gram-positive organisms such as *L. lactis* consists in a cell wall made of peptidoglycan grafted with proteins, teichoic acids, lipoteichoic acids and polysaccharides [14], [15]. Cell wall anchored proteins account among important factors that have been shown to drive interactions of Gram-positive bacteria with various biotic or abiotic surfaces [16], [17]. This has been extensively studied in pathogens [16] and to a lesser extent in lactococci [18], [19]. Proteins harboring a C-terminal anchoring domain featuring an LPxTG-like motif (in which x may be any amino acid) form an important type of surface proteins in Gram-positive bacteria [20]. These LPxTG proteins are secreted across the plasma membrane by the Sec-dependent pathway and are subsequently processed by transpeptidases termed sortases [21], [22], [23], [24]. LPxTG substrates may have different destinies depending on their structural characteristics. In one case, LPxTG substrates are processed by an ubiquitous cysteine transpeptidase termed housekeeping sortase or class A sortase (SrtA) that cleaves the Thr-Gly bond within the LPxT^▾^G motif and forms another isopeptide bond between the resulting C-terminal Thr carboxyl group and an amino group in the interpeptide bridge of the peptidoglycan precursor lipid II [25]. The archetype of such substrates is protein A from *Staphylococcus aureus* whose sortase-mediated anchoring mechanism was the first to be characterized at the molecular level [26], [27]. Similar SrtA machineries have been functionally analyzed in several Gram-positive pathogens in which they are involved in anchoring proteins associated with virulence [28], [29], [30], [31], [32], [33]. A functional sortase A is also present in the non pathogenic bacterium *L. lactis* and is involved in the anchoring of several proteins that play an important role in the biology of *L. lactis*, for example the cell wall anchored proteinase that allows growth of lactococci in milk [34]. While this SrtA-driven anchoring mechanism leads to cell wall anchoring of proteins as monomers, some LPxTG proteins harboring defined additional amino acids motifs have a different destiny and may polymerize into a pilus anchored at the cell surface of Gram-positive bacteria.

Pili of Gram-positive bacteria have first been characterized in pathogens in which they are involved in adhesion and virulence. Their formation was discovered by pioneering works in *Actinomyces naeslundii* [35], [36] and *Corynebacterium diphtheriae* [37], and later in *Bacillus cereus* [38], *Bacillus anthracis* [39], *Enterococcus faecalis* [40], *Enterococcus faecium* [41], and species of the genus *Streptococcus* including *S. agalactiae* [22], [42], *S. pneumoniae* [43], *S. pyogenes* [44], [45], and *S. suis* [46]. Pili are proteinaceous appendages of 1–10 nm in diameter protruding 2–3 µm outside of bacterial cells. In contrast to pili found in Gram-negative bacteria that are polymers of noncovalently bound subunits, those in Gram-positive bacteria are most often heteropolymers formed by 2 or 3 LPxTG proteins termed pilins that are covalently polymerized by transpeptidation reactions catalyzed by pilus-specific class C sortases (SrtC) [47]. Once assembled, the pilus fibers may be anchored to lipid II either by SrtA [48], [49] or by SrtC [50]. Typically, one of these pilins is called the backbone pilin as 100 to 200 of these subunits are assembled head-to-tail to form the pilus shaft [51]. The 1 or 2 other pilins are termed ancillary pilins and can be either located exclusively at the cap and at the base of the pilus, respectively, or spread along the pilus shaft [52]. In addition to their LPxTG-like anchoring motif, backbone pilins almost always contain 2 consensus motifs *i.e.* a WxxxVxVYPK pilin motif and an YxLxETxAPxGY E-box motif, so called because of the presence of a highly conserved glutamic acid residue (E) [37], [53]. As for the pilin motif, the lysine residue (K) is involved in the covalent linkage of one backbone subunit with the threonine residue (T) in the LPxTG motif of the next backbone subunit [54]. The function of the E-box motif is not essential for assembly of the pilus backbone but has been proved to be essential in incorporation of ancillary pilins into the pilus and in formation of intramolecular bond that occur in backbone pilins [51], [53]. In contrast with backbone pilins, pilin and E-box motifs seem to be dispensable for ancillary pilins raising the question of the underlying mechanism for their incorporation into the pilus backbone. As for the cap pilin, it can be linked through its LPxTG-like motif to the pilin motif of the adjacent backbone pilin. In the case of the basal pilin, it has been shown that a lysine residue, albeit not embedded within a recognizable pilin motif, is involved in an amide bond with the LPxTG of the neighbor backbone pilin [55].

Until recently, pili have not been observed in non-pathogenic Gram-positive bacteria. However, the presence of pili and related adhesion properties were recently reported in some probiotic bacteria including *Lactobacillus rhamnosus* GG and members of the genus *Bifidobacterium* [56], [57], [58], [59], [60]. In the LAB *L. lactis*, we recently provided genetic and biochemical evidence of a housekeeping sortase A able to anchor at least 5 LPxTG proteins to its cell wall [34]. The study also revealed the presence of a sortase C gene within a putative pilus gene cluster raising the possibility of pilus biogenesis in this bacterium. Although lactococcal pili have never been reported, a study in which *L. lactis* was used as a host for expression of components of a *S. agalactiae* pilus operon showed that *L. lactis* could drive heterologous pilus biogenesis when *S. agalactiae* sortase C gene was expressed along with the components of the streptococcal pilus operon. In contrast, over-expression of the sole *S. agalactiae* pilin genes failed to enable production of pili in *L. lactis* indicating that autochthonous lactococcal sortase C did not polymerize *S. agalactiae* pilins into pili [3], [61]. This observation raised the question of lactococcal sortase C functionality and/or of its expression level under the retained experimental conditions.

In the present work, we characterized the *L. lactis* pilus gene cluster and studied its function in pilus assembly. The contribution of the different components of the cluster is reported and we show that this yet unreported trait in lactococci provides this bacterium with a different lifestyle both in liquid medium and on solid surfaces, inducing bacterial auto-aggregation and reticulated biofilms, respectively.

## Results

### *L. lactis* IL1403 Harbors a Putative Class C sortase Locus Containing all the Genes for Pilus Biogenesis

Two putative sortases have been previously described in *L. lactis* IL1403, YlcC (NP_267269; designated as SrtA), and YhhA (NP_266915; designated as SrtC) [21], [34], [62]. The 432 residues SrtC protein harbors the TLVTC sortase signature at positions 221–225, which includes the catalytic cystein residue and the two essential His and Arg residues [63], at positions 163 and 234, respectively. The SrtC precursor harbors putative N- and C-terminal transmembrane domains. *In silico* analyses predict no cleavage of the N-terminal signal sequence suggesting that SrtC might be retained at the membrane by both its N- and C-termini [64], [65].

**Figure 1:**
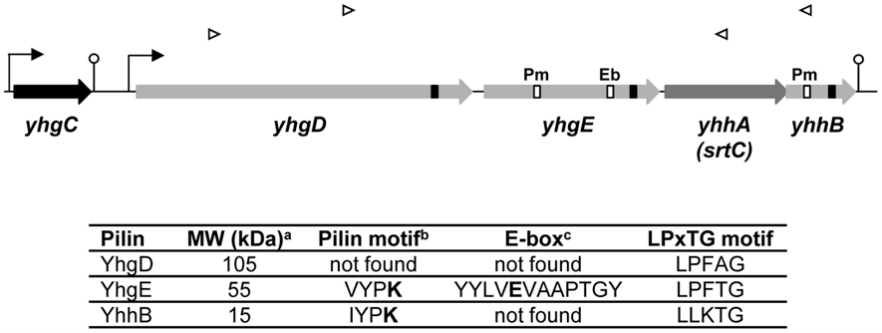
The *srtC* genomic locus in *L. lactis* IL1403. Gene names are indicated. Putative promoters and transcriptional terminators are schemed. Open triangles represent oligonucleotides that allowed cDNA amplification and RT-PCR experiments (see text). The gene products of *yhgD*, *yhgE*, *yhhB* (filled in light gray) all encode LPxTG motif (represented as black boxes)-containing proteins suggesting that they are substrates of SrtC (filled in darker gray). Some also harbor pilin motif (Pm) and E-box (Eb) motif (white boxes) indicated in the central table. The *yhgC* gene (filled in black) encodes a protein whose C-terminus shows homology with Rgg/GadR/MutR-type transcription regulators [92], [108]. Note that the *yhhB* sequence contained a G743T substitution compared to the sequence deposited at the NCBI. ^a^, Theoretical molecular weight corresponding to mature proteins (precursor proteins devoid of both signal sequence and CWA domain); ^b^, the putative lysine residue (K) that is essential in pilin oligomerization is marked in bold; ^c^, the putative glutamic acid residue (E) of the E-box is marked in bold.

The *srtC* locus also comprises *yhgD*, *yhgE*, and *yhhB* genes that encode proteins with an LPxTG-like cell wall anchoring domain, a known feature of both sortase A and C substrates (Fig. 1). YhgD, YhgE, and YhhB also contain consensus sequences found in pilin proteins [66] *i.e.* an N-terminal signal sequence in all 3 precursor proteins, a VYPK pilin motif in YhgE and YhhB, and an E-box motif in YhgE only (Fig. 1). These observations suggested that *L. lactis* harbors all the components necessary for pilus biogenesis and that YhgE that shows both pilin and E-box motifs could be the backbone pilin [37]. While no function has yet been assigned to these putative pilins, a search for functional domains revealed that YhgE harbors two Cna protein B-type domains described in the *Staphylococcus aureus* collagen-binding surface protein [67]. Aside from other *Lactococcus* strains, a homology search did not yield matches neither to yet characterized pilins nor to other proteins. Analyses of the *srtC* locus in other *L. lactis* strains showed same gene organization and high protein identity in strains MG1363 (NC_009004), KF147 (NC_013656), CV56 (NC_017486), and IO-1 (AP012281) while the *srtC* locus of *L. lactis* SK11 (NC_008527) and A76 (NC_017492) showed the presence of an IS element within *yhgC*, the gene just upstream of *yhgD*, and important deletions in *yhgD*, *srtC*, and *yhhB* genes.

### The Predicted Pilus Gene Cluster is Organized and Expressed as an Operon

Sequence analyses of the pilus gene cluster predict an operon structure for *yhgD*, *yhgE*, *srtC*, and *yhhB* (Fig. 1). RT-PCR analysis was performed using primer pairs designed to generate overlapping amplicons on mRNA encoded by the four genes, and a couple of primers designed to detect an mRNA extending to *yhgC*, the gene just upstream of *yhgD* (Fig. 1). When cDNA obtained with a primer that binds to *yhhB* was used as template, amplicons of the expected sizes were obtained with primer pairs hybridizing at *yhgD*-*srtC* and *yhgD*-*yhhB*. In contrast, no amplification was obtained with primers binding *yhgC*-*yhgD*. These results indicate that *yhgD*, *yhgE*, *srtC*, and *yhhB* are co-transcribed in *L. lactis* IL1403 under the growth conditions used and that *yhgC* does not belong to this operon. Such an operon structure in which the pilin structural genes are co-transcribed along with a class C sortase gene has also been reported in *Enterococcus* sp. [40], [68], and is referred to below as the *pil* operon.

### Functionality of the Pilus Biogenesis Machinery in *L. lactis*

**Figure 2:**
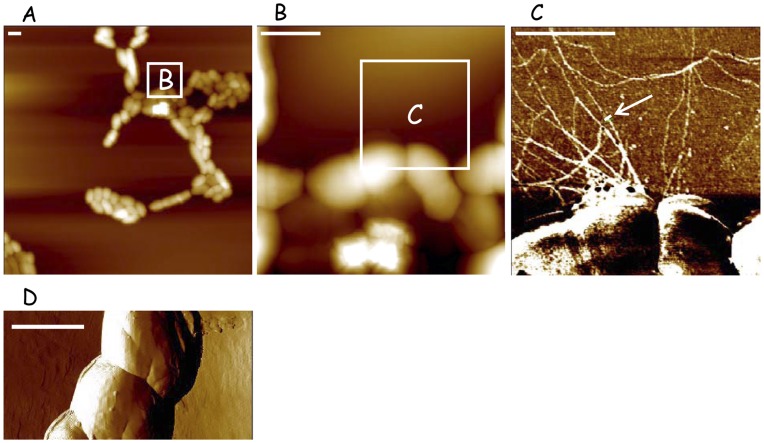
Observation by AFM of lactococcal pili in *L. lactis* IL1403 over-expressing the *pil* operon. A and B are topographic representations at two scales, C is the deflection representation obtained from the framed part of B, D corresponds to the control *L. lactis* IL1403 strain harboring pIL253 plasmid (Table 1). A white bar shown by an arrow in C indicates where the pilus diameter was measured assuming radial symmetry and flattening. (Scale bars, 1 µm).

The ability of the *pil* operon to drive pilus biogenesis in *L. lactis* was assessed by western blot analyses of cell wall protein extracts using antibodies raised against the pilins YhgD, YhgE, and YhhB. No signals were detected, suggesting that under our culture conditions, the structural genes for these proteins are expressed at low level or these proteins are produced at levels undetectable by western blot (data not shown). To favor pilin expression, the *pil* operon (*yhgD*-*yhgE*-*srtC*-*yhhB*) was cloned under the control of the constitutive lactococcal promoter P23 [69] in the high copy number vector pIL253 [70] to yield the pPil plasmid. Analysis of wild type *L. lactis* IL1403 over-expressing the entire *pil* operon (designated IL pPil) by atomic force microscopy (AFM) revealed the presence of filamentous structures on the bacterial surface that were frequently tangled, with several fibers wrapping around each other (Fig. 2A–C) and that were absent in the wild type strain (Fig. 2D). The fibers reached up to 3 µm in length and around 5 nm in width (Fig. 2C). Interestingly, these pili were only present at distinct foci on the bacterial surface and as observed in other studies [40], [71], [72], only part of the bacterial population seemed piliated. High-magnification AFM examination revealed thin fibers with bulbous decorations suggesting the presence of special proteinaceous structures as described in *S. pneumoniae* pili [71]. These results suggest that the *pil* operon encodes a functional pilus biogenesis machinery in *L. lactis*.

### Pilin Subunits in *L. lactis* Pilus Architecture

**Figure 3:**
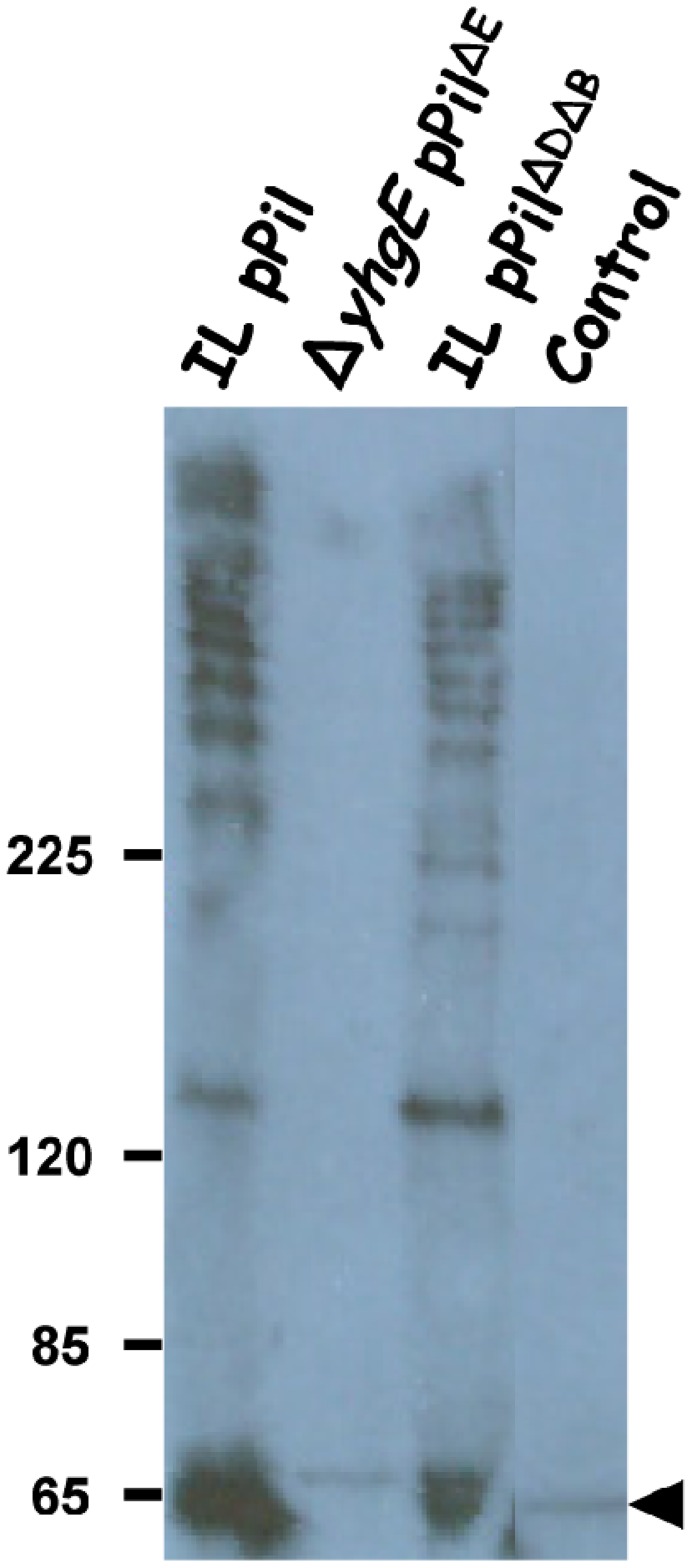
Western blot analysis of cell wall-anchored proteins of *L. lactis* strains using anti-YhgE antibodies. Equivalent protein amounts from *L. lactis* control strain and from derivatives expressing all or parts of the *pil* operon were separated on 3–8% gradient Tris-acetate Criterion XT SDS-PAGE gel and were detected by immunoblotting. Control refers to *L. lactis* IL1403 strain harboring pIL253 plasmid. For strain designation, see Table 1. The positions of molecular mass standards (in kilodaltons) are indicated and the YhgE monomer is shown by a black arrowhead.

To characterize lactococcal pili, cell wall protein extracts from the IL pPil strain were analyzed by western blotting using polyclonal antibodies raised against YhgE, the putative backbone pilin. The antibodies revealed a pattern of discrete ladder-like bands typical of pilin-type proteins [53]. The ladders seem to correspond to multiples of the lowest 65 kDa band (Fig. 3). The theoretical molecular weight of YhgE being 55 kDa, the detected ∼65 kDa band might correspond to YhgE linked to fragments of peptidoglycan. As YhgE is rich in threonine residues, which are common sites for glycosylation, it is also possible that its modified electrophoretic mobility is due to glycosylation. To confirm that the detected signals corresponded to YhgE, a cell wall extract from a Δ*yhgE L. lactis* chromosomal mutant constitutively expressing the *pil* operon lacking *yhgE* (Δ*yhgE* pPil^ΔE^, *i.e.* VE17190, see Table 1) was analyzed. No signal was detected by the anti-YhgE antibodies, thus confirming the specificity of YhgE signals seen above (Fig. 3). To assess the role of the other pilins encoded by the *pil* operon in YhgE polymerization, cell wall extracts were analyzed from the *L. lactis* strain VE17183 (Table 1), in which the plasmid over-expresses the *pil* operon deleted of *yhgD* and *yhhB* genes (pPil^ΔDΔB^). The profile revealed by anti-YhgE antibodies was similar to that obtained from the IL pPil strain expressing the complete *pil* operon (Fig. 3) suggesting that pilins YhgD and YhhB are not required for polymerization of YhgE.

**Table 1:** Bacterial strains and plasmids used in this study.

Strain or plasmid	Relevant characteristic(s)*	Reference
**Strain**		
*E. coli*		
	*E. coli* TG1 (Invitrogen)	[97]
*L. lactis* IL1403		
VE57034	Wild-type (WT), plasmid-free strain	[109]
VE17061	WT, pIL253, Em^r^ (control)	This study
VE17173	WT, pVE5618, Em^r^ (IL pPil)	This study
VE17148	WT, pVE5585, Em^r^ (IL pPil^ΔD^)	This study
VE17183	WT, pVE5621, Em^r^ (IL pPil^ΔDΔB^)	This study
*L. lactis* Δ*srtC*		
VE5760	*srtC* deleted *L. lactis* IL1403 (Δ*srtC*)	This study
VE17191	Δ*srtC*, pVE5624, Em^r^ (Δ*srtC* pPil*)	This study
*L. lactis* Δ*srtA*		
VE5775	*srtA* deleted *L. lactis* IL1403 (Δ*srtA*)	[34]
VE17174	Δ*srtA*, pVE5618, Em^r^ (Δ*srtA* pPil)	This study
VE5802	Δ*srtA,* p*srtA*, Tet^r^ (Δ*srtA* p*srtA*)	[34]
VE17176	Δ*srtA,* p*srtA*, pVE5618, Em^r^ Tet^r^ (Δ*srtA* p*srtA* pPil)	This study
*L. lactis* Δ*yhgE*		
VE17187	*yhgE* deleted *L. lactis* IL1403 (Δ*yhgE*)	This study
VE17190	Δ*yhgE*, pVE5623, Em^r^ (Δ*yhgE* pPil^ΔE^)	This study
*L. lactis* isolates		
2276-89	isolated from blood	[110]
2885-86	isolated from a wound	[110]
2742-86	isolated from blood	[110]
1585-85	isolated from blood	[110]
1385-85	isolated from blood	[110]
1384-85	isolated from blood	[110]
810-85	isolated from eye	[110]
868-78	isolated from urine	[110]
KF147	isolated from mung bean	[111]
KF282	isolated from cress	[111]
NCDO2118	Isolated from frozen peas	C. Delorme$
**Plasmid**		
PCR®II-Blunt-TOPO (pTOPO)	ColE1, Kan^r^	Invitrogen
pIL253	pAMβ1, Em^r^	[70]
pG^+^host9	Ts derivative of pWV01, Em^r^	[112]
pDelB1	1497 bp upstream of *srtC* cloned into pG^+^host9, Em^r^	This study
pDelB	1497 bp upstream of *srtC* and 997 bp downstream of *srtC* cloned into pG^+^host9, Em^r^	This study
p*srtA*	pIL2608::*srtA*, Tet^r^	[34]
pVE5618	pIL253::*P23-yhgD-yhgE-srtC-yhhB*, Em^r^ (pPil)	This study
pVE5585	pIL253::*P23-yhgE-srtC-yhhB*, Em^r^ (pPil^ΔD^)	This study
pVE5621	pIL253::*P23-yhgE-srtC*, Em^r^ (pPil^ΔDΔB^)	This study
pVE5623	pIL253::*P23-yhgD-srtC-yhhB*, Em^r^ (pPil^ΔE^)	This study
pVE5624	pIL253::*P23-yhgD-yhgE-srtC* * *-yhhB*, Em^r^ (pPil*)	This study
pVE5615	pIL253::*P23::emm6*, Em^r^	This study
pVE5619	pIL253::*P23*, Em^r^	This study
pVE16009	pTOPO::*yhgD-yhgE-srtC-yhhB*, Kan^r^	This study
pVE16030	1540 bp downstream of *yhgE* cloned into pTOPO, Kan^r^	This study
pVE16031	1603 bp upstream of *yhgE* cloned into pTOPO, Kan^r^	This study
pVE5329	1659 bp of pVE16031 cloned into pVE16030, Kan^r^	This study
pVE16021	pG^+^host9::pVE5329, Kan^r^ Em^r^	This study

* ColE1 and pAMβ1 refer to the replicon; Tet^r^, tetracycline resistance; Em^r^, erythromycin resistance; Kan^r^, kanamycin resistance; srtC*, mutated *srtC* gene encoding an inactive sortase C; plasmid and strain designations used in the text are indicated in parentheses.

$ Christine Delorme, INRA, Micalis-UMR1319, F78350-Jouy-en-Josas.

**Figure 4:**
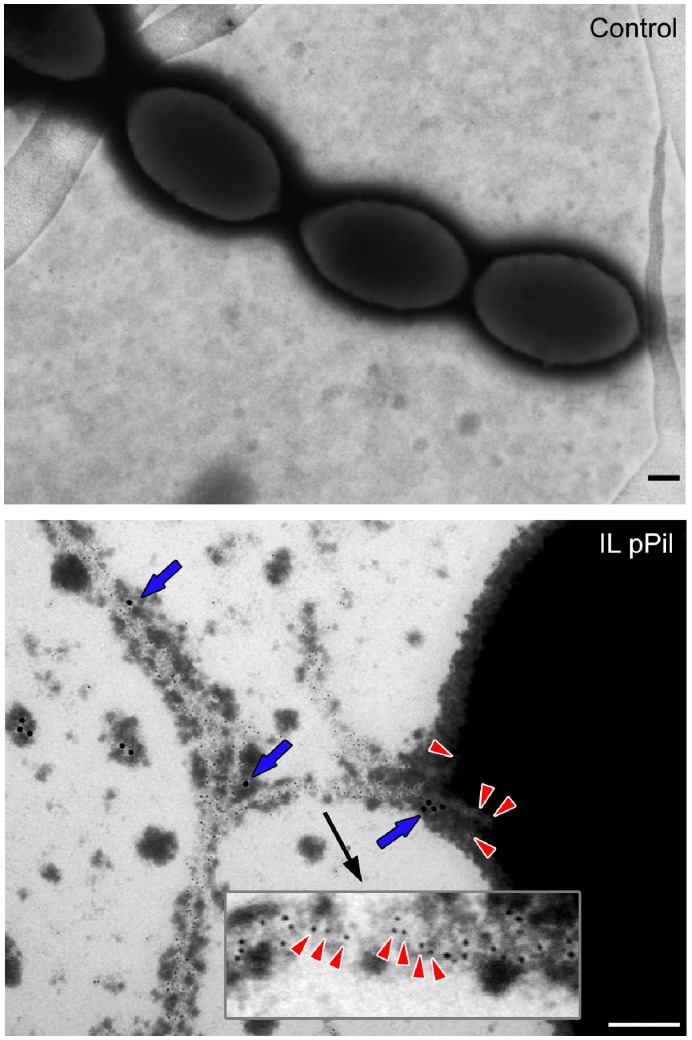
Immunolocalization of the YhgE backbone pilin by TEM. Negative staining of *L. lactis* strains was performed using phosphotungstic acid. Strains were immobilized on Formvar-carbon-coated nickel grids and pilins were detected using as primary antibodies, guinea-pig anti-YhgE and rabbit anti-YhgD polyclonal antibodies. The preparations were treated with secondary antibodies consisting of anti-guinea-pig conjugated to 5 nm gold beads for YhgE and anti-rabbit conjugated to 15 nm gold beads for YhgD. Red arrowheads, YhgE pilin subunits present in the pilus fibers or in the cell wall; blue arrows, YhgD pilin subunits. Control refers to *L. lactis* IL1403 strain harboring pIL253 plasmid and IL pPil to *L. lactis* IL1403 strain in which the *pil* operon is overexpressed (Table 1). (Scale bars, 200 nm).

To further assess the composition of lactococcal pili, we performed a double immunogold labeling experiment, after which cells were visualized using transmission electron microscopy (TEM) and scanning electron microscopy (SEM). *L. lactis* IL1403 pPil bacteria were stained with guinea-pig anti-YhgE polyclonal antibodies and/or rabbit anti-YhgD polyclonal antibodies followed by gold labeled anti-guinea-pig and/or gold labeled anti-rabbit secondary antibodies, conjugated to gold beads of different size. For TEM, a negative staining treatment for pilus fiber visualization was also applied on native bacteria or on bacteria that had been immunogold-labeled. Observation by TEM showed long pilus fibers up to 3 µm (Fig. S1). Immunogold labeling with anti-YhgE antibodies revealed signals all along the pilus structure and on the cell surface (Fig. 4). In contrast, YhgD staining appeared as isolated spots located on the cell surface and possibly at the pilus tip (Fig. 4). This dual localization of pilins has already been observed in other studies [73], [74] and can be explained by the presence of an LPxTG motif in pilins that makes them potential substrates for monomeric cell wall anchoring by the housekeeping sortase SrtA [75]. Overall, these observations confirmed that YhgE constitutes the major pilin whose polymerization forms the pilus backbone. In contrast, the localization of YhgD within the pilus appeared uncertain.

**Figure 5:**
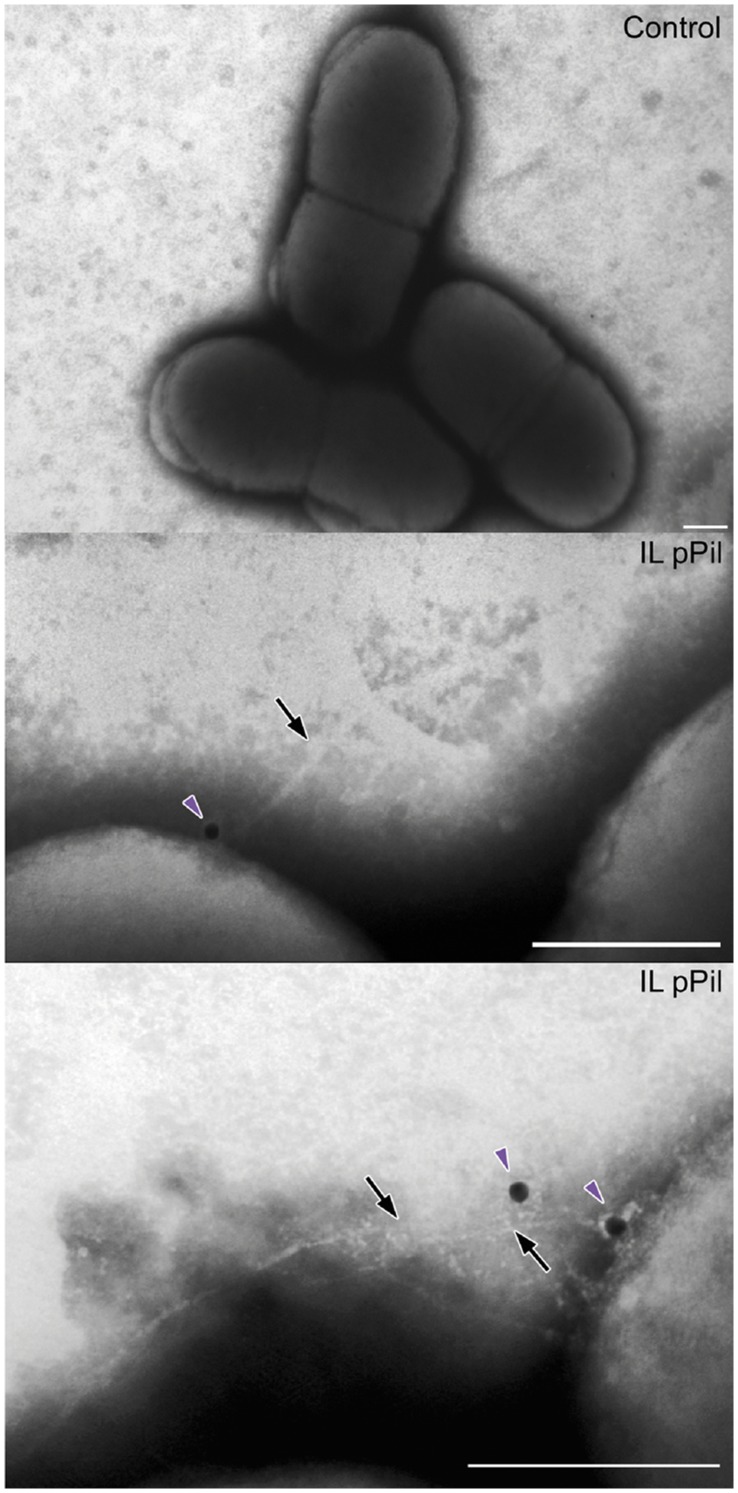
Immunolocalization of the YhhB basal pilin. Negative staining of *L. lactis* strains was performed using phosphotungstic acid. Strains were immobilized on Formvar-carbon-coated nickel grids and pilins were detected using as primary antibodies, guinea-pig anti-YhhB polyclonal antibodies. The preparations were treated with secondary antibodies consisting of anti-guinea-pig conjugated to 15 nm gold beads. The negatively stained pili are indicated by black arrows and the YhhB pilin is indicated with purple arrowheads. Control refers to *L. lactis* IL1403 strain harboring pIL253 plasmid and IL pPil to *L. lactis* IL1403 strain in which the *pil* operon is overexpressed (Table 1). (Scale bars, 200 nm).

Experiments as described above were performed to assess the contribution of YhhB to pilus structure. Upon YhhB immunogold labeling and negative staining, we could visualize YhhB present at single foci at the base of pili (Fig. 5). In contrast, we were unable to demonstrate the presence of YhhB in cell wall protein extracts of *L. lactis* pPil by western blot analysis (data not shown). This is possibly due to low level of YhhB produced and to the detection threshold in the western blot system used as this has also been reported with other ancillary pilins [60].

**Figure 6:**
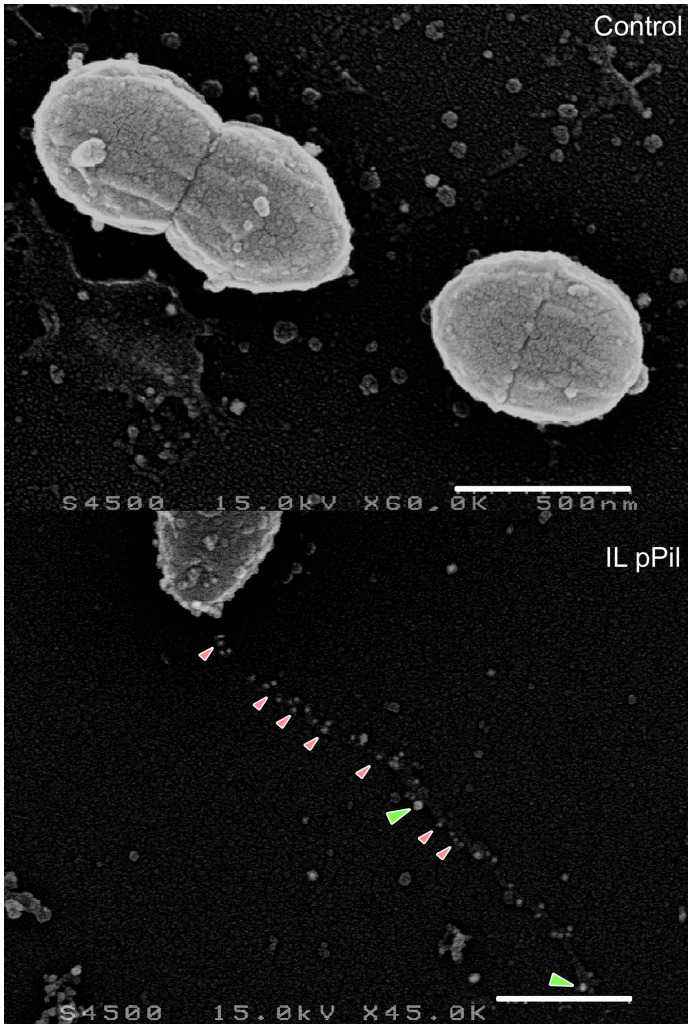
Immunolocalization of the YhgE shaft pilin and of the YhgD cap pilin by SEM. Fixed bacteria were applied to glass cover slips and stained with primary antibodies consisting of guinea-pig anti-YhgE and rabbit anti-YhgD polyclonal antibodies. Preparations were further incubated with colloidal-gold-conjugated secondary antibodies anti-guinea-pig-15 nm gold beads and anti-rabbit-25 nm gold beads. The backbone (YhgE) and cap (YhgD) pilin subunits are indicated with small pink and large green arrowheads, respectively. Control refers to *L. lactis* IL1403 strain harboring pIL253 plasmid and IL pPil to *L. lactis* IL1403 strain in which the *pil* operon is over-expressed (Table 1). (Scale bars, 500 nm).

To further examine the distribution of YhgE and YhgD pilins along the pilus fiber, SEM analysis was performed following double YhgE and YhgD immunogold labeling. As expected, YhgE was detected all along the pilus backbone (Fig. 6). The YhgD pilin appeared to localize at the pilus tip although another YhgD subunit was sometimes also detected in the core of the pilus fiber (Fig. 6). Most pilus fibers visualized in *L. lactis* pPil strains appeared tangled, as already indicated (Fig. 2). Interestingly, YhgD pilin subunits were detected at knobs corresponding to intersection points where distinct pilus fibers associated (data not shown). This suggests that YhgD is the pilus cap pilin and that it might be involved in interaction mechanisms between *L. lactis* pili.

### Components Involved in Pilus Polymerization and Cell Wall Anchoring

**Figure 7:**
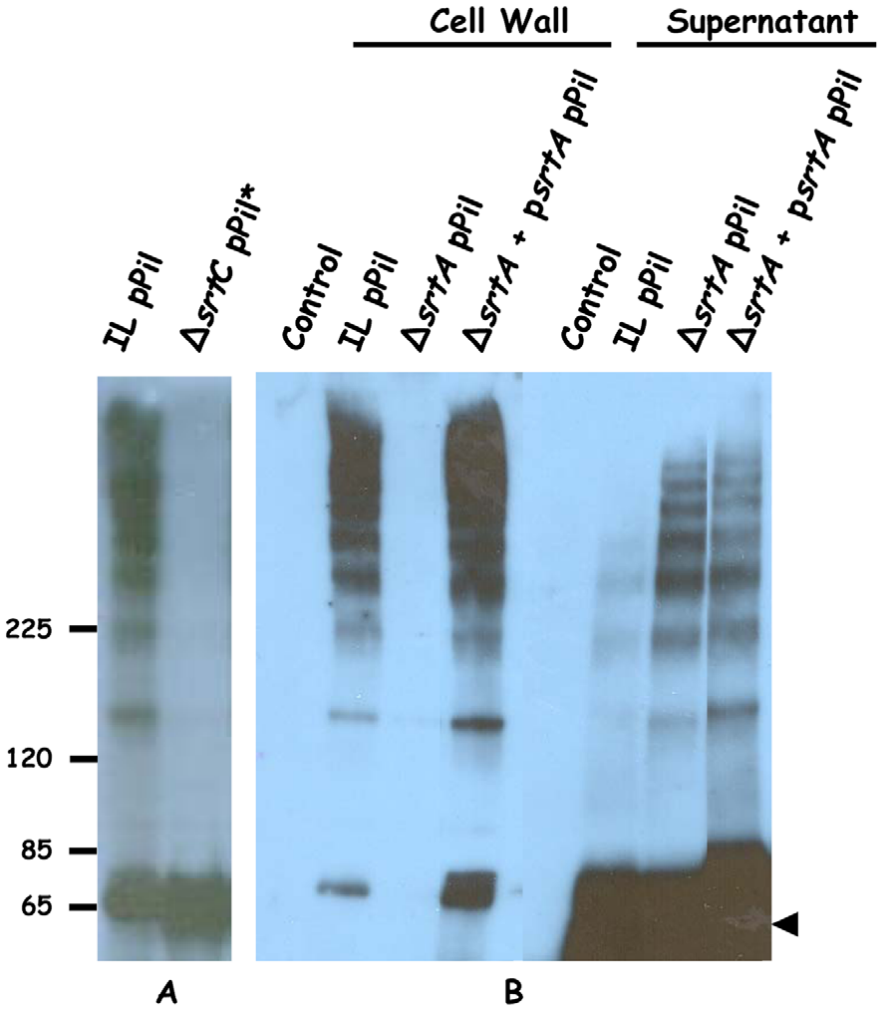
Distribution of YghE in cell wall and supernatant fractions of *L. lactis* strains. Equivalent protein amounts from control strain and of derivatives expressing all or parts of the *pil* operon were separated on 3–8% gradient Tris-acetate Criterion XT SDS-PAGE gel and were detected by immunoblotting. A, Analysis of cell wall-anchored proteins; B, Analysis of both cell wall-anchored proteins and of supernatant-released proteins. Control refers to *L. lactis* IL1403 strain harboring the pIL253 plasmid. For strain designation, see Table 1. The positions of molecular mass standards (in kilodaltons) are indicated and the YhgE monomer is indicated by a black arrow.

To determine the role of sortase C in pilus biogenesis in *L. lactis*, a plasmid harboring the *pil* operon with an inactivated *srtC* gene (yhgD-yhgE-srtC*-yhhB) was constructed. This inactivated *srtC* gene consisted in the substitution of the essential active site cysteine for an alanine residue. The plasmid was established in a *srtC* chromosomal deletion mutant, thus resulting in a *L. lactis* strain totally devoid of sortase C activity (designated Δ*srtC* pPil^*^). Western blot analysis of cell wall extracts from this strain using anti-YhgE antibodies revealed a unique band at ∼ 65 kDa corresponding to the monomer of YhgE (Fig. 7A). However, the ladders corresponding to oligomers of YhgE were not detected. This result indicates that SrtC is responsible for the polymerization of YhgE. The presence of the monomer of YhgE in the cell wall also suggests that SrtA is able to anchor YhgE to the cell wall since it is the only sortase present in the bacteria in the absence of SrtC. This corroborates previous analyses in which we observed that the anchor domain of YhgE fused to a reporter protein was processed by SrtA [34].

To further investigate the role played by SrtA in pilus assembly in *L. lactis,* the entire *pil* operon was over-expressed in a Δ*srtA* chromosomal deletion mutant. Western blot analysis using anti-YhgE antibodies revealed that the ∼ 65 kDa and high molecular weight (HMW) bands detected in the *L. lactis* IL1403 pPil strain were absent in the cell wall extracts of the Δ*srtA* pPil strain (Fig. 7B). Complementation of this mutant using the p*srtA* plasmid [34] encoding a functional *srtA* gene restored and even increased the attachment of pili at the bacterial surface as shown by the presence of the ladders detected by anti-YhgE antibodies in cell wall extracts of the complemented strain (Fig. 7B). Since sortases are not involved in the export of their substrates, we hypothesized that in the absence of SrtA, YhgE would still be processed by SrtC but the oligomers would be released in the culture supernatant. Indeed, analysis of the culture supernatants of different strains showed that the YhgE ladders observed in the cell wall fraction of *L. lactis* IL1403 pPil was found in the supernatant of the Δ*srtA* pPil strain (Fig. 7B). This result showed that *L. lactis* Δ*srtA* pPil was still able to form polymers of YhgE but had lost the ability to attach the pili to its cell wall indicating that sortase A is responsible for this function. Interestingly, Δ*srtA* pPil strain complemented with the p*srtA* plasmid not only restored pilus anchoring at the cell surface but also released pili in the supernatant. We believe that over-expression of both *srtA* and the *pil* operon produces high amount of SrtA-pili intermediates. Besides an increase of the amount of pili attached at the cell surface (Fig. 7B), we propose that in the presence of an excess of SrtA-pili intermediates, lipid II, the substrate on which SrtA attaches LPxTG proteins [76], becomes a limiting factor [77]. This results in the release of SrtA-pili intermediates in the supernatant. Altogether, these results demonstrate that in *L. lactis* the pilus backbone is formed by the polymerization activity of SrtC independently of cell wall anchoring, which is mediated by the housekeeping sortase SrtA. To assess specific contribution of ancillary pilins to the cell wall anchoring of pili, the same type of western analysis was performed with cell wall and supernatant extracts from *L. lactis* IL pPil, pPil^ΔD^ or pPil^ΔDΔB^ using anti-YhgE antibodies. The majority of YhgE polymers were detected in the cell wall fraction of *L. lactis* IL pPil and pPil^ΔD^ strains while they appeared to be released in the supernatant fraction of *L. lactis* IL pPil^ΔDΔB^ (data not shown). This indicates that YhhB is involved in cell wall anchoring of the pilus to the cell wall. This is in agreement with our above observation using immunogold labeling analyses in which YhhB was shown to be localized at the base of the pilus.

### Occurrence of Pili in *L. lactis* Isolates

To assess pili production in natural *L. lactis* isolates, a few strains isolated from clinical or vegetal environments were analyzed for pili display. Following negative staining and TEM analysis, 2 out of 8 *L. lactis* clinical isolates and 2 out of 3 vegetal isolates showed, under standard laboratory growth conditions, some pili at their surface (Fig. S2). The number of pili detected varied greatly from few pili in strains 2885-86, 810-85, and NCDO2118 to high pili number in strain KF282 isolated from cress (Table 1). This suggests that in contrast to the model strain *L. lactis* IL1403, some lactococci are able to produce pili under the retained laboratory conditions. To assess genetic organization of the *pil* operon in those strains, PCR analyses followed by DNA sequence analyses were performed using primer pairs matching 2 different genes in the *pil* operon. The obtained results (data not shown) indicated a similar genetic organization of the *pil* operon in *L. lactis* IL1403 and in the natural lactococci strains studied (similar gene order, presence of a single *srtC* gene, and high level of DNA sequence identity).

### Involvement of Pili in Auto-aggregation and Biofilm Formation

#### Bacterial auto-aggregation mediated by the *pil* operon

**Figure 8:**
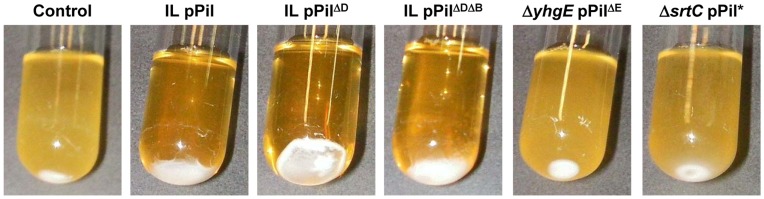
Auto-aggregation phenotype of *L. lactis* cultures. Strains over-expressing all or parts of the *pil* operon as indicated above the pictures were grown overnight under static conditions. Control refers to *L. lactis* IL1403 strain harboring the pIL253 plasmid. For strain designation, see Table 1.

In the course of this work, we observed that some of the genetic constructs expressed in *L. lactis* IL1403 induced an auto-aggregation phenotype in liquid cultures. To test whether pili contribute to this phenotype, a macroscopic study was performed using bacterial cultures of the constructed *L. lactis* strains. Overnight liquid cultures showed that the control *L. lactis* IL1403 strain remained mainly as a planktonic suspension and produced a small pellet (Fig. 8), while the IL pPil strain exhibited a clear auto-aggregation phenotype. The same phenotype was observed in the strain over-expressing only *yhgE* and *srtC* genes (*via* IL pPil^ΔDΔB^), indicating that YhgD and YhhB are dispensable for the auto-aggregation phenotype. In contrast, cultures of Δ*yhgE* pPil^ΔE^ (lacking *yhgE*) or of Δ*srtC* pPil^*^ (lacking active *srtC*) failed to auto-aggregate. YhgE and SrtC therefore appear to be the key players in the observed auto-aggregation phenotype. These observations and the roles played by YhgE and SrtC in pilus biogenesis suggest that the auto-aggregation phenotype is mediated by the pili.

#### Biofilm formation

**Figure 9:**
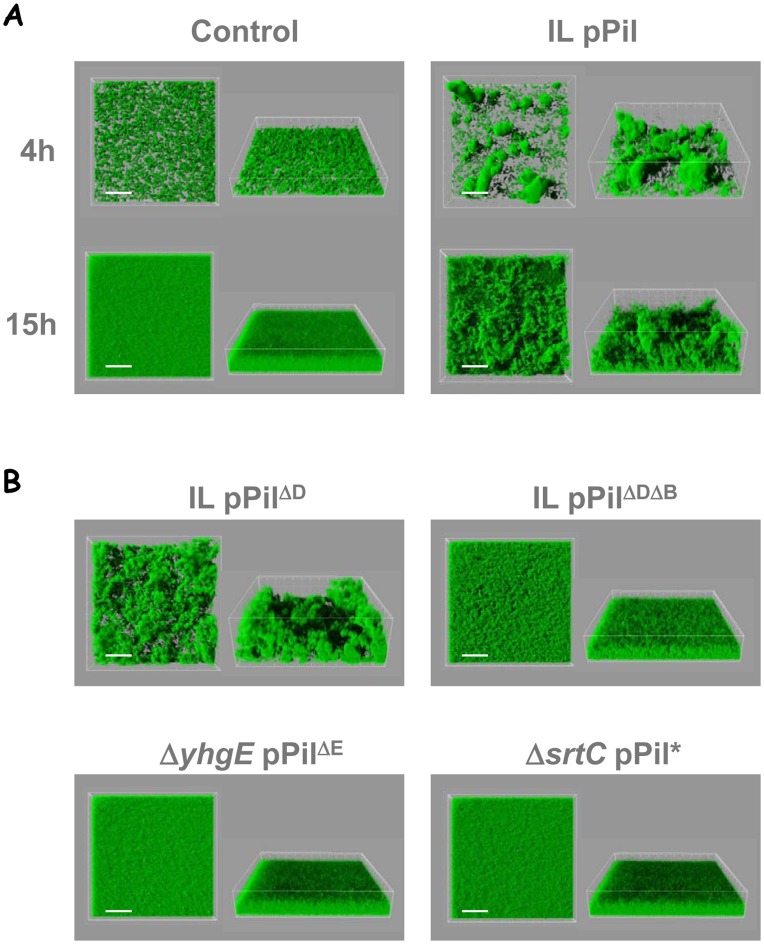
Three-dimensional biofilm structures of *L. lactis* IL1403 strains obtained from confocal image series. Control *L. lactis* IL1403, IL pPil, and derivative strains were analyzed after 4 and 15 h growth in microtiter plates at 30°C under static conditions. Image analyses were performed using IMARIS software. A, 4 and 15 h biofilms; B, 15 h biofilms. For strain designation, see Table 1. (Scale bars, 50 µm).

**Figure 10:**
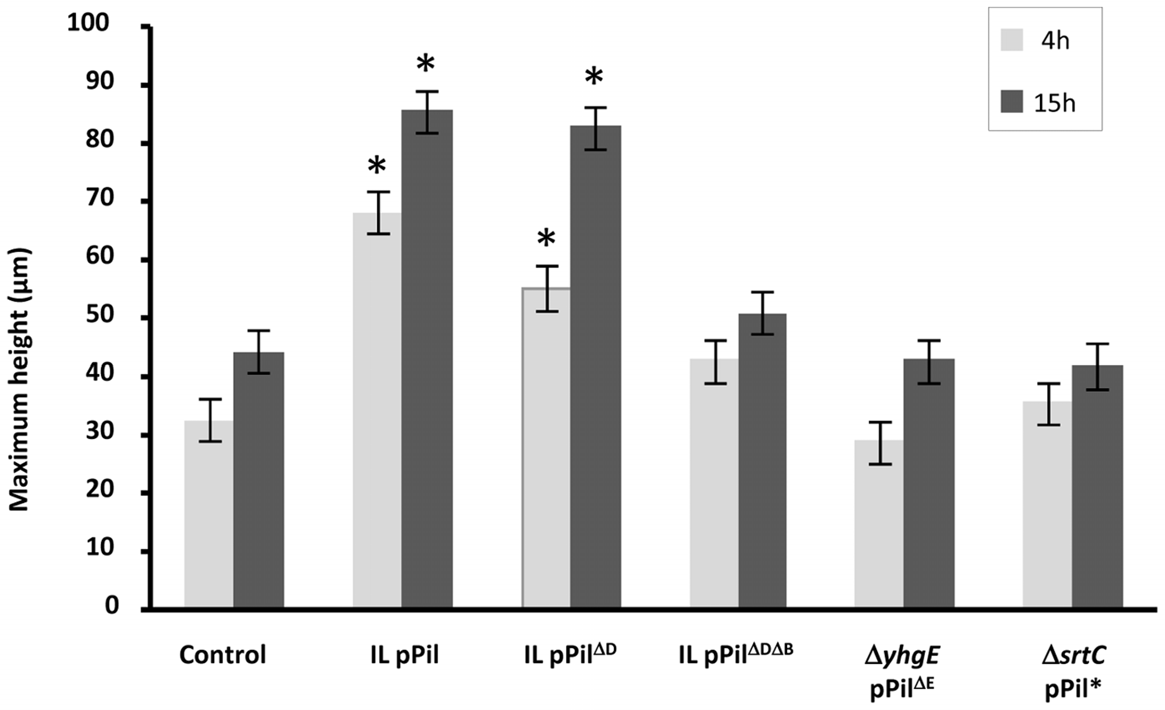
Maximum height of biofilms obtained with *L. lactis* strains. Strains that yielded biofilms whose maximum height measured at 4 and 15 h of growth was significantly different (*P*<0.05) to that of control *L. lactis* IL1403 are marked by asterisks. Standard error is indicated. For strain designation, see Table 1. Indicated values are the mean of 3 determinations per experiment.

To assess whether the presence of pili on the cell surface of *L. lactis* influenced its capacity to form biofilms on solid surfaces, the three-dimensional structure of lactococcal biofilms was studied using confocal laser scanning microscopy (CLSM). Lactococcal biofilms were observed at 4 and 15 h of growth and images corresponding to three-dimensional reconstructions from confocal stack images are shown in Fig. 9 and Fig. S3. As described for *L. lactis* MG1363 [78], the control *L. lactis* IL1403 strain quickly developed a compact and uniform biofilm on the surface (Fig. 9A). In contrast, the pili-displaying IL pPil strain expanded from clustered bacteria that were distributed as patches on the surface. Over time, confluence of these bacterial patches formed highly reticulated biofilms that appeared heterogeneous, rough and aerial (Fig. 9A). In an attempt to assign this reticulated biofilm phenotype of piliated bacteria to specific pilin proteins, the different *L. lactis* strains constructed were further analyzed. When either *yhgE* (Δ*yhgE* pPil^ΔE^) or *srtC* (Δ*srtC* pPil*) was omitted, the observed biofilms appeared similar to those obtained with the control *L. lactis* IL1403 strain (Fig. 9A and B) indicating that pili are responsible for the aspects of the biofilm produced by the IL pPil strain. In contrast, *L. lactis* IL pPil^ΔD^ showed the same reticulated biofilm as the IL pPil strain suggesting that YhgD is dispensable for this phenotype (Fig. 9B). Interestingly, when both *yhgD* and *yhhB* were missing in the over-expressed operon, the resulting biofilms of *L. lactis* IL pPil^ΔDΔB^ lost their patchy and aerial structure while they remained rougher than the biofilm of the control *L. lactis* IL1403 strain (Fig. 9A and B). To examine further structural parameters of the above observed biofilms, the maximum height of biofilms was quantified from CLSM image series. This revealed that *L. lactis* IL pPil showed a maximum height that was significantly higher than that of the control *L. lactis* IL1403 strain (Fig. 10, *P*<0.05). The same observations were made for the IL pPil^ΔD^ strain (*P*<0.05). In contrast, strains that over-expressed an operon lacking *yhhB*, *yhgE* or an active *srtC* gene showed biofilms with heights comparable to that of the control *L. lactis* IL1403 strain (*P*>0.05). Attempts to correlate those various phenotypes (Table 2) show that while the cap pilin YhgD is dispensable for pili display, auto-aggregation phenotype, and reticulated biofilm, the basal pilin YhhB is involved in pili display and reticulated biofilm phenotype.

**Table 2:** Pili display and related auto-aggregation and biofilm phenotypes in *L. lactis* IL pPil and derivatives.

Strain*	Pili display	Auto-aggregation	Reticulated biofilms
IL pPil	_+_	+	+
Δ*srtC* pPil*	−	−	−
IL pPil^ΔD^	+	+	+
Δ*yhgE* pPil^ΔE^	−	−	−
IL pPil^ΔDΔB^	−	+	−

* for strain designation, see Table 1.

## Discussion

Study of pilus biogenesis in Gram-positive bacteria is relatively recent compared to Gram-negative species. Most of the studies conducted so far have focused on pathogens because of the role often played by pili in virulence [79]. Pili are long filaments displayed on bacterial surface and due to their adhesive properties are involved in the first stages of host colonization [47]. In Gram-positive pathogens, study of the mechanism of pilus assembly and the functions of pili in the genera *Actinomyces, Bacillus, Corynebacterium*, *Enterococcus* and *Streptococcus* has been considerable in recent years [22], [37], [38], [39], [40], [41], [42], [43], [44], [45], [46], [80]. The ultimate objectives of these studies are to find new drug targets and to characterize pili components as new vaccine candidates [52], [81]. In LAB, reports on the presence of pili are rare. It is only recently that pili were characterized in *Lactobacillus rhamnosus* GG [58], [60] and were shown to drive mucosal adhesion allowing gastrointestinal tract colonization by this probiotic bacterium [58], [82]. In spite of substantial research efforts on the biology of *L. lactis*, another LAB, pili have never been described in this bacterium. In a previous study we reported the identification of two putative sortase genes in the genome of *L. lactis* and characterized one of them as encoding the housekeeping class A sortase that anchored LPxTG proteins to the cell wall [34]. The other sortase, a class C sortase, did not appear to be involved in cell wall anchoring of the studied LPxTG proteins (data not shown) and this prompted us to study its possible function.

The pilus biogenesis mechanism dissected in *L. lactis* can be schemed as a 2-step process *i.e.* polymerization of pilin subunits and cell wall anchoring of the nascent pilus.

### Polymerization of Pilin Subunits

All the components for pilus assembly in *L. lactis* were found to form an operon structure comprising 3 pilin genes and one sortase C gene. Upon constitutive over-expression of the *pil* operon, the pili detected at the surface of *L. lactis* were formed by pilin polymerization catalyzed exclusively by SrtC since a single amino acid substitution in the active site of SrtC abrogated pilin polymerization. This confirms the function of class C sortases as pilus-dedicated sortases [24]. *L. lactis* pili consist of polymerized YhgE that forms the backbone pilin, YhgD that corresponds to the cap pilin and YhhB that is the pilus base pilin. These respective localizations of pilins are in line with the genetic organization of the *pil* operon (*yhgD-yhgE-yhhA-yhhB*) since YhgD whose structural gene is the first in the operon, would be the first pilin to be translated and exported through the membrane, forming the tip of the growing pilus. The respective localizations of pilins in the pilus are also consistent with the specific motifs detected in each pilin. YhgE is the only pilin that contains both a pilin and an E-box motifs, 2 features that appear crucial for most backbone pilins [83]. As for YhhB, the presence of a pilin motif would allow transpeptidation with an LPxTG motif of the adjacent YhgE pilin while its own LPxTG motif would be involved in an isopeptide bond with the peptidoglycan precursor lipid II. YhgD which appeared devoid of a consensus pilin motif could only be engaged at the tip of the pilus through an isopeptide bound between its LPxTG motif and the Lys residue present in the pilin motif of the adjacent YhgE pilin. However, few YhgD pilin subunits were also present in the core of the pilus fibers. Observations of ancillary pilins being localized at various loci in the pilus fibers have also been made in other studies of Gram-positive pili [37], [48], [60], [71] while the structural studies performed so far concluded on an exclusive localization of the 2 pilins at the tip and at the base of the pilus, respectively [55], [61], [84]. In the case of YhgD, its apparent presence in the core of the pili might be a consequence of the observed tangling of pili or might result from limitations inherent to immunogold labeling studies. Alternatively, we cannot exclude that YhgD is indeed assembled within the pilus backbone as some pilins devoid of a recognizable pilin motif were shown to be polymerized through a lysine residue that did not lie within a pilin motif [51], [55].

### Tethering of the Pilus Fibers to the Cell Wall

The linkage of the nascent pilus fibers to the cell wall relies on the housekeeping sortase A of *L. lactis* since a single *srtA* gene deletion mutant released the majority of pili in the medium. This indicates that SrtC is unable to catalyze this step in pilus assembly. The same observation was made in some other Gram-positive bacteria [48], [49], unlike in *S. pneumoniae* in which a class C sortase is also implicated in cell wall anchoring of pili [50]. Another essential component in cell wall tethering of the pilus fiber is the basal pilin YhhB as deletion of its structural gene induced the release of pili in the medium. This function of YhhB could require a proline-rich tail (PPNKPKPAPKPIEVPKAP) located before the LPxTG sorting motif of this pilin. Such tails forming a polyproline-II (PPII)-like helix were recently identified in other basal pilins and are believed to facilitate cell wall anchoring of pilus fibers [55]. Our observation that the absence of YhhB induces pili release in the medium suggests that the backbone pilin YhgE is not efficiently processed by SrtA in the transpeptidation reaction between the LPxTG motif of YhgE and an amino acid residue of the peptidoglycan precursor lipid II. Probably, SrtC has a higher affinity for YhgE than SrtA does, raising the yet unanswered question of the determinants of substrate specificity of sortases. Interestingly, *L. lactis* pili appeared in distinct foci. This raises the question of the underlying mechanism allowing pili deposition at discrete foci on the cell surface. There is increasing evidence that pilin secretion, processing, and polymerization as well as the anchoring of the pilus to lipid II intermediate take place in the vicinity of the division septum at a unique microdomain of the bacterial membrane termed the ExPortal [72], [85], [86], [87]. This focal localization of sortases and LPxTG proteins involves a protein signature consisting of a positively charged domain flanking a transmembrane helix [72]. Such domain is present in sortases A and C of *L. lactis* IL1403 as well as in pilins YhgD, YhgE, and YhhB suggesting that a similar phenomenon takes place during pilus biogenesis in *L. lactis*. The mechanism for further distribution of the pili from the septum to distinct foci of the cell surface is unclear. As the pili of *L. lactis* are anchored to the cell wall by sortase A, a distribution pattern similar to that proposed for sortase A substrates could occur. This includes colocalization of SecA, SrtA and LPxTG substrates at the division septa where peptidoglycan synthesis occurs and distribution at discrete surface foci as the peptidoglycan expands [72], [86], [88].

Biofilm formation is a complex process that comprises several distinct stages. The same bacterial species can form biofilms of different architectures depending on various factors including growth media, cell surface physicochemical properties and type of surface on which biofilm is built. Pili are known to be involved in biofilm formation in both Gram-positive and Gram-negative bacteria [89], [90]. Pili, by their adhesive properties can mediate early attachment to surfaces and cell to cell interaction leading to auto-aggregation and ultimately to biofilm formation [91]. In this study, we have shown that *L. lactis* cells that display pili induce auto-aggregation in liquid cultures and formation of thicker biofilms than the wild-type strain. Since YhgE is the major pilin in *L. lactis*, we believe that it promotes the auto-aggregation of lactococcal cells and ultimately the thicker biofilm. We did not identify any adhesive domain in the YhgE sequence. However, YhgE contains a Cna-B domain at its C-terminal region. This domain is found in the *Staphylococcus aureus* collagen-binding surface protein. Structural analyses have suggested that it forms a stalk in *S. aureus* collagen-binding proteins that positions the collagen-binding domain ideally for interaction with its ligand [67]. Cna-B domains are also present in the pilus tip pilin PilA of GBS in which they flank a von Willebrand adhesion domain [48]. These are evidences pointing to the existence of adhesion domains in the sequence of YhgE. These domains remain however to be identified.

Pili detected at the surface of *L. lactis* IL1403 were only observed when the *pil* operon was constitutively expressed from a plasmid. It is possible that under the growth conditions we used, the expression of the pilus operon is too low for the pili to be detected by our methods of analysis. One could speculate that when *L. lactis* is placed under a suitable environment, regulatory factors promote transcriptional activation of the operon and/or a stabilization of the messenger leading to production and display of pili at the bacterial surface. In this regard, it would be interesting to test a possible involvement of the *yhgC* gene located upstream of the pilus operon that encodes a Rgg-type regulator [92]. It would also be worth looking for growth conditions and/or culture media in which expression of *pil* operon and production of pili is favored.

This work represents the first comprehensive study of pilus biogenesis in a non-pathogenic model organism. Using a wide range of approaches including molecular biology, immunochemistry, and imaging, we demonstrated the functionality of the pilus gene cluster of *L. lactis* IL1403 and showed the respective contribution of lactococcal sortases and pilins in pilus biogenesis. This observation made on a model laboratory *L. lactis* strain was validated in natural *L. lactis* isolates in which pili were detected. This corroborates another current study performed in a vegetal *L. lactis* isolate which displays pili at its surface (Chapot-Chartier, personal communication). Altogether, these results indicate that pili production is a spread trait in natural and non modified lactococci strains. Dairy *L. lactis* strains might have evolved towards reduced biosynthetic capacities as they adapted to new nutrient-rich environment such as milk. This has been observed for other traits such as carbohydrate catabolic capacities and amino acid prototrophy that are higher in strains derived from vegetal environments than in dairy strains [93], [94], [95]. Since pili greatly modify the lifestyle of lactococci, our present knowledge of the interaction of lactococci with its environment, often acquired with dairy, pili-devoid laboratory strains, should be revisited. In some applications in which *L. lactis* is or could be used, such as food fermentations and probiotics, pili might be important players in interaction of lactococci with food matrices or with components of the gastrointestinal tract.

## Experimental Procedures

### Bacteria, Plasmids, and Media

Bacterial strains and plasmids used in this study are listed in Table 1. Static cultures of *L. lactis* were prepared in M17 [96] containing 0.5% glucose, or in Brain Heart Infusion (Difco, Detroit, MI) media at 30°C unless otherwise mentioned. *Escherichia coli* were grown in LB (Luria-Bertani) broth supplemented with thymine at 37°C upon shaking [97]. The following antibiotics were used as required: for *E. coli*, erythromycin (150 µg/mL), and kanamycin (50 µg/mL); for *L. lactis*, erythromycin (5 µg/mL), kanamycin (400 µg/mL), and tetracycline (5 µg/mL).

### General DNA Manipulation and Transformation Procedures

General molecular biology techniques were performed essentially as described [97]. Plasmids were purified from *E. coli* and *L. lactis* as described [98], [99]. Plasmids were introduced into *L. lactis* by electroporation [100] and into *E*. *coli* by the heat shock method [97]. Restriction and modification enzymes were purchased from New England Biolabs (Ipswich, MA, USA). PCR primers were purchased from Eurogentec (Angers, France) and are listed in Table S1. All genetic constructs were verified by DNA sequencing.

### RNA Extraction

Total RNAs were extracted from 20 mL of bacterial cultures collected at the exponential growth phase (OD_600 nm_∼0.5) and at the beginning of the stationary phase (OD_600 nm_∼1.5). Cells were harvested by a rapid centrifugation (6 000 *g*, 1 min) and suspended in 400 µL TE buffer (10 mM Tris-HCl, pH 8.0, 1 mM EDTA) and 500 µL phenol-chloroform 5∶1 (v:v) before being transferred into 1.5 mL microtubes containing 0.5 g of 0.1 mm diameter glass beads (Sigma Aldrich, Saint Louis, MO, USA), 15 µL of 20% SDS and 30 µL of 3 M sodium acetate (pH 4.8). Cells were disrupted using a Fast Prep FP120 system (Bio1001; Thermo Electron Corporation) at maximum speed for 40 seconds. After centrifugation at 13 000 *g* for 20 min at 4°C, the aqueous phase was recovered, and RNA was purified with 500 µL chloroform: isoamyl alcohol (v:v). Total RNA was purified using the High Pure RNA Isolation kit (Roche, Mannheim, Germany) according to manufacturer’s protocol. Extracts were adjusted to about 0.2 or 0.5 µg/µL to perform rigorous DNase treatments using a DNA-free kit (Ambion, Austin, TX, USA) according to the manufacturer’s instructions. RNA concentration and purity were assessed by the *A*260 nm/*A*230 nm and *A*260 nm/*A*280 nm ratios, respectively [101] using a NanoDrop ND-1000 Spectrophotometer (NanoDrop Technologies, Wilmington, DE, USA).

### Reverse Transcription - PCR

Reverse transcription was processed from 4 µg of total RNA. RNA was treated at 65°C during 5 min to denature RNA secondary structures and annealed with specific primer yhhB2 or PrSorB4 (Table S1) during 10 min at 20°C. cDNA was synthesized by a 10 h reverse transcription PCR at 42°C using Primescript Reverse transcriptase (Clontech, Ozyme, Saint-Quentin-en-Yvelines, France) and UltraPure dNTP (Clontech), followed by enzyme inactivation (15 min at 70°C). PCR reactions using the cDNA as template were performed with yhgC1/yhgD2, PrSorA2/PrSorD3 and yhgD6/yhhB3 primer pairs derived from the *srtC* locus sequence (Table S1). The absence of genomic DNA in RNA samples was checked by PCR using the RNA extracts as template.

### Construction of Unmarked Deletion of the srtC Gene in L. lactis IL1403

The *srtC* gene (*yhhA*) of *L. lactis* IL1403 was deleted using the pG^+^host-based allelic exchange method [102].

#### Construction of the plasmid used for *srtC* deletion

To construct the *srtC*-deleting plasmid, the 5′ flanking region was amplified using primers SrtB1/SrtB2 (Table S1). The PCR product was treated with *Xho*I and *Cla*I and cloned into pG^+^host9 restricted with *Nsi*I-*Eco*RV to yield the pDelB1 plasmid (Table 1). The 3′ flanking region was amplified with primers SrtB3/SrtB4 (Table S1) and the product was digested with *Spe*I and *Sac*II, and then cloned into pDelB1 treated with *Nsi*I-*Eco*RV to yield the pDelB plasmid (Table 1).

#### Construction of a Δ*srtC* chromosomal mutant

The pDelB plasmid was established in *L. lactis* IL1403. The resulting strain was grown overnight at 30°C in the presence of erythromycin. Culture dilutions were plated onto erythromycin containing BHI plates and incubated 48 hours at 37°C (a non-permissive temperature for pG^+^host9 replication). The erythromycin-resistant colonies obtained harbored the pDelB plasmid integrated in the chromosome, resulting in the duplication of the region upstream and downstream of *srtC* gene. Selected integrants were grown overnight at 37°C in BHI broth containing erythromycin. Cultures were then diluted 1/100 in BHI without erythromycin and grown at 30°C to OD_600_ ∼0.8. At this temperature the replication origin of pG^+^host9 is active which favors plasmid excision. To eliminate the excised plasmid, dilutions of the cultures were plated onto BHI plates lacking erythromycin, and incubated at 37°C. Strain in which *srtC* was deleted was identified by both PCR using primers that flanked *srtC* locus genes and Southern blot analysis. The obtained mutant (VE5760, Table 1) showed bacterial morphology and growth kinetic similar to those of wild type strain (data not shown).

### Construction of an In-frame Unmarked Deletion of the yhgE Gene in L. lactis IL1403

The deletion of the *yhgE* gene was performed essentially as described above for *srtC* gene deletion.

#### Construction of the plasmid used for *yhgE* deletion

The chromosomal regions upstream and downstream of the *yhgE* gene were PCR-amplified with primer pairs yhgD3/yhgDE and yhgE8/yhhB4, respectively (Table S1). The 1603 bp and 1540 bp products were cloned into pCR®II-Blunt-TOPO® vector (Invitrogen, Cergy Pontoise, France) to yield pVE16031 and pVE16030 (Table 1), respectively. The 1659 bp *Xho*I fragment from the pVE16031 plasmid was cloned into the *Xho*I site of the pVE16030 plasmid to yield the pVE5329 plasmid (Table 1). Finally, the *Sac*II pVE5329 fragment, containing the *yhgE* flanking regions, was cloned into the *Sac*II site of the pG^+^host9 yielding pVE16021 (Table 1).

#### Construction of the in-frame *yhgE* chromosomal deletion mutant

The Δ*yhgE* mutant was obtained following the same procedure as that for *srtC* chromosomal deletion, except that M17 glucose medium was used with both erythromycin and kanamycin for selection. Strain in which *yhgE* was deleted was identified by both PCR using primers that flanked the *yhgE* gene and by genomic DNA sequencing. The obtained mutant (VE17187, Table 1) showed bacterial morphology and growth kinetic similar to those of wild type strain (data not shown).

### Construction of a Plasmid for Over-expression of the Entire Pil Operon

Plasmid pVE5212 [99] was cut with *Sac*I and self-ligated to remove the pBluescript part. The resulting pVE5615 plasmid (Table 1) was digested with *Eco*RV and *Sma*I, self-ligated and established into *L. lactis* leading to pVE5619 (Table 1), a ready to use pIL253::*P23* plasmid. The full length *pil* operon (from *yhgD* to *yhhB*) was PCR amplified from genomic DNA of *L. lactis* IL1403 using primers yhgD9/yhhC3 (Table S1) and Phusion® High-Fidelity DNA Polymerase (Finnzymes, Ozyme, Saint-Quentin-en-Yvelines, France). A novel *Apa*I restriction site was introduced by PCR, just at the 5′ of the ATG start codon of *yhgD*. The 7818 bp PCR product was sub-cloned into pCR®II-Blunt-TOPO® plasmid (Invitrogen) to create pVE16009 (Table 1). The resulting plasmid was digested with *Apa*I and *Xho*I and the 8187 bp fragment was ligated into pVE5619 cut with the same enzymes resulting in pIL253::*P23*::*yhgD*-*yhgE*-*srtC*-*yhhB* (pVE5618, Table 1) designated pPil.

### Construction of Plasmids for Over-expression of Derivatives of the Pil Operon

#### Construction of pIL253::*P23*::*yhgD*-*srtC*-*yhhB* plasmid

To create an in-frame deletion of the *yhgE* gene in pVE5618 obtained above, the upstream and downstream chromosomal regions of the *yhgE* gene were PCR-amplified with the primer pairs yhgD1/yhgDE1 and yhhA9/yhhA10, respectively (Table S1). The 2 obtained amplicons along with primers yhgD1/yhhA10 were used in an overlap PCR [103], [104] to generate a portion of the *pil* operon deleted of the *yhgE* gene. The obtained amplicon was cloned into the pCR®II-Blunt-TOPO® vector (Invitrogen) before being released as a 791 bp *Drd*I-*Pci*I fragment that was cloned into the *Drd*I*-Pci*I pVE5618 plasmid to yield pVE5623 designated pPil^ΔE^ (Table 1).

#### Construction of plasmid pIL253::*P23*::*yhgD*-*yhgE*-srtC*-*yhhB* incorporating a Cys/Ala substitution of the SrtC cystein catalytic residue

Substitution of the essential C225 residue of SrtC to an alanine residue was achieved using splicing by overlap extension PCR [104]. The upstream and downstream regions of the cystein residue in *srtC* gene were PCR-amplified with the primer couples yhhA6/yhhA7 and yhhA8/pVE5618a (Table S1), respectively. The two PCR products were used as templates for the overlap extension PCR using primers yhhA6/pVE5618a. The amplicon was cloned into pCR®II-Blunt-TOPO® vector, then released as a 1837 bp *Xho*I-*Pci*I fragment which was sub-cloned into the *Xho*I-*Pci*I digested pVE5618 plasmid to yield pVE5624 designated pPil* (Table 1).

#### Construction of pIL253::*P23*::*yhgE*-*srtC*-*yhhB* plasmid

Portion of the *pil* operon from *yhgE* gene to *yhhB* gene was amplified from *L. lactis* IL1403 genomic DNA using primers yhgE9/yhhC3 (Table S1). The yhgE9 primer includes an *Apa*I site upstream of the ATG start codon of *yhgE*. The 4143 bp amplified product was digested with *Apa*I and cloned into the 5093 bp *Apa*I-*Sma*I fragment of pVE5615 to yield pVE5585 designated pPil^ΔD^ (Table 1).

#### Construction of pIL253::*P23*::*yhgE*-*srtC* plasmid

Plasmid pVE5585 was digested by *Sal*I and *Blp*I, modified using Kleenow large fragment enzyme (New England Biolabs) and T4 DNA polymerase (New England Biolabs) and self-ligated to yield pVE5621 designated pPil^ΔDΔB^ (Table 1).

### Production of Anti-pilin Antibodies

Portion of genes *yhgD*, *yhgE*, and *yhhB* encoding the three putative pilin proteins, were PCR-amplified from *L. lactis* IL1403 genomic DNA using primer pairs yhgD8/yhgD13, yhgE1/yhgE2 and yhhB1/yhhB2, respectively (Table S1). Purified PCR products were cloned into *E. coli* expression vector pET200/D-TOPO (Invitrogen) for *yhgD* and pET101/D-TOPO (Invitrogen) for *yhgE* and *yhhB*, to produce 6xHis-tagged recombinant proteins. The obtained plasmids were transformed into *E. coli* Top10 or BL21 codon+ (for vector containing portion of the *yhhB* gene). For both YhgE and YhhB, recombinant proteins were purified with Econo-Pac columns (BioRad, Marnes-la-Coquette, France) according to the manufacturer’s instructions and used for custom antibody production in guinea-pigs (Centre de Production Animale, Olivet, France). For YhgD recombinant protein, a SDS-PAGE protein band at the expected size, was verified by MALDI-TOF analysis at the PAPPSO platform (http://pappso.inra.fr/) and used for custom antibody production in rabbits (Covalab, Villeurbanne, France). Polyclonal antibodies were produced according to the immunization protocol of the manufacturers.

### Extraction of Cell Wall Anchored and Culture Supernatant Proteins and Western Blot Analyses

To study pilin monomers and polymers in *L. lactis* strains, the extraction method described by Garandeau *et al*. [29] and used by Konto-Ghiorghi *et al*. [48] to study pili in *S. agalactiae* was adopted. Overnight bacterial cultures (10 mL) were centrifuged at 2 300 *g* for 10 min. Proteins from 1.6 mL of the culture supernatants were obtained by precipitation using trichloroacetic acid (TCA) at 16% final concentration. After 20 min on ice, samples were centrifuged for 15 min at 4°C and 15 000 *g*. Pellets were resuspended in 8 µL per OD_600_ unit of 50 mM NaOH [99] and an equal volume of 2×loading buffer [105] was added. To prepare cell wall protein fractions, the bacterial pellets from the above overnight culture were resuspended in 500 µL of 4% SDS −0.5 M Tris-HCl pH 8. The bacterial suspensions were boiled for 10 min and then centrifuged at 2 300 *g* for 5 min. The pellets were washed once in 0.5 M Tris-HCl pH 8 and resuspended in 100 µL per OD_600_ unit in 1×loading buffer. When protein concentration was to be measured, the BioRad protein assay (BioRad) was used following the manufacturer’s instructions. All protein extracts were boiled 10 min and equal amounts of proteins were separated in 3–8% gradient SDS-PAGE gels (Criterion XT Tris-acetate, BioRad). Polyvinylidene difluoride membranes (Millipore, Billerica, MA, USA) were used for electrotransfer and they were blocked in PBS containing 10% milk (w:v) for 2 h. Pilin proteins were detected using specific primary polyclonal antibodies (see below) and horseradish peroxidase-coupled specific secondary antibodies. Detection was performed using the Immobilon Western Chemiluminescent HRP Substrate (Millipore) according to the manufacturer’s recommendations.

### Atomic Force Microscopy Imaging of Bacteria

Bacteria were grown overnight in tubes or on plates, washed in Phosphate Buffered Saline (PBS) and fixed in 3% paraformaldehyde (PFA) in PBS during 20 min. Fixed bacteria were successively washed in PBS and in distilled water. Bacterial samples were spotted onto freshly cleaned glass microscope slides and allowed to air-dry at room temperature in a dust-free environment. Surface topology of these bacteria was characterized by Atomic Force Microscopy (AFM) in contact mode (PicoSPM, Molecular Imaging, ScienTec, Palaiseau, France) operating under air at 22°C. For these experiments, we used a cantilever (silicon nitrides gold-coated oxide-sharpened, ScienTec, Palaiseau, France) with a spring constant of ∼ 0.38 N.m^-1^. Topographic, deflection and friction images were acquired at a scanning rate of 1 line/s and for 512 lines/image.

### Immunogold Labeling and Electron Microscopy

Two electron microscopy methods, transmission electron microscopy (TEM) and scanning electron microscopy (SEM), were used. Bacteria from overnight cultures were harvested and washed in PBS before fixation with PFA 3% during 30 min at room temperature.

For TEM, fixed bacteria were harvested, and resuspended in PBS. Formvar-carbon-coated nickel grids were floated on drops of fixed bacteria. For SEM analyses, fixed bacteria were directly applied to glass cover slips. For both techniques, after 20 min at room temperature, samples were washed twice in PBS for 5 min and submitted to three consecutive 5 min incubations in PBS supplemented with NH_4_Cl 50 mM followed by three PBS washes. Standard immunology procedure was followed: briefly, samples were blocked with PBS 1% BSA 0.1% BSAc (Aurion, Wageningen, The Netherlands) for 30 min, and incubated for 2 hours with one or two of the following primary antibodies, *i.e.* the YhgE guinea-pig, the YhgD rabbit polyclonal antibodies, and the YhhB guinea-pig antibodies. Preparations were washed and incubated for 1 h with the conjugated to colloidal gold secondary antibodies, the guinea-pig conjugated to gold beads (British BioCell, Cardiff, UK or Aurion, Wageningen, The Netherlands) and the rabbit conjugated to gold beads (Aurion, Wageningen, The Netherlands). Samples were washed with PBS.

For TEM, samples were fixed in PBS containing 2.5% glutaraldehyde for 5 min before consecutive washes in PBS and in distilled water. Negative staining was performed using phosphotungstic acid. Examination was made using a Zeiss EM902 electron microscope operated at 80 kV (Carl Zeiss – France), and images were acquired with a charge-coupled device camera (Megaview III) and analysed with ITEM Software (Eloïse, France) at the MIMA2 Platform (http://mima2.jouy.inra.fr).

For SEM, samples were fixed overnight in PBS containing 1% glutaraldehyde, washed in PBS and then incubated in 50% ethanol during 15 min. Bacteria were dehydrated through a graded series of ethanol (from 50 to 100%) followed by critical point drying with CO_2_. Dried specimens were sputter coated with palladium, with a GUN ionic evaporator PEC 682 and were examined and photographed at the MIMA2 platform (http://mima2.jouy.inra.fr) with a Hitachi S-4500 Scanning Electro Microscope field emission scanning electron microscope operating at 5 kV.

### Biofilm Formation Assays

Biofilms structural dynamic of *L. lactis* strains in M17 medium supplemented with 0.5% glucose and erythromycin on polystyrene 96-well microtiter plates (Greiner Bio-one, France) with µclear® base (Polystyrene, thickness of 190±5 µm) were studied as described previously [106]. Briefly, overnight cultures were used to inoculate the growth medium to an OD_600 nm_ of 0.01 and 250 µL of those adjusted cultures were dispensed into the wells. After 1 h of adhesion at 30°C, the liquid was delicately removed and refilled with 250 µL of sterilized medium. The plates were incubated at 30°C for 4 or 15 hours. Biofilms were stained during 15 min with M17 containing 5 µM Syto 9 (Invitrogen), a cell permeant green fluorescent nucleic acid marker. The stained biofilms were observed using CLSM (Leica SP2 AOBS, LEICA Microsystems, France) at the MIMA2 microscopic platform (http://mima2.jouy.inra.fr). All biofilms were scanned at 400 Hz using a 10× or a 63× water immersion objective lens. Fluorescently stained bacteria were excited at 488 nm with an Argon laser beam, and the emitted fluorescence collected in the range 500–600 nm on a photomultiplier. The assay was performed in two independent experiments, each in duplicate for each strain and stacks were acquired at different area in each well. Image analysis was performed using IMARIS 7.0 software (Bitplane, Switzerland) to reconstruct three-dimensional projections of biofilm structures. Maximum height of biofilms was extracted from CLSM images series using the Leica LITE software. Statistical analyses (one-way ANOVA) were performed using Statgraphics v6.0 software (Manugistics, Rockville, USA). Significance was defined as a *P* value associated with a Fisher test value lower than 0.05.

### In silico Analyses

Search for sortase homologs was performed using BLAST (www.ncbi.nlm.nih.gov//genomes/geblast.cgi?gi=171). Analyses of DNA for open reading frames were performed using the EasyGene 1.0 server of the Center for Biological Sequence Analysis (http://www.cbs.dtu.dk/services/EasyGene/). Promoter prediction was conducted using the PPP software from the MolGen bioinformatics webtools (http://bioinformatics.biol.rug.nl/web_tools.html). Signal peptide prediction and cleavage site prediction were performed with SignalP4.0 [64]. Transmembrane helices were predicted using the TMHMM server [107]. Search for conserved domains was performed using KEGG database (http://www.genome.jp/kegg/).

## Supporting Information

Figure S1 **TEM images of negatively stained** ***L. lactis*** **strains.** Control refers to *L. lactis* IL1403 strain harboring pIL253 plasmid and IL pPil to *L. lactis* IL1403 strain in which the *pil* operon is over-expressed. Pili are indicated by black arrows. (Scale bars, 500 nm).
(TIF)

Figure S2 **TEM images of negatively stained** ***L. lactis*** **strains.** Pili are indicated by black arrows. Strain designation is indicated at the up and right side of the images (see Table 1). (Scale bars, 200 nm).
(TIF)

Figure S3 **Three-dimensional biofilm structures of** ***L. lactis*** **strains obtained from confocal image series.** Control *L. lactis* IL1403, IL pPil, and derivative strains were analyzed after 4 and 15 h growth in microtiter plates at 30°C under static conditions. Three images per strain are presented. Image analyses were performed using IMARIS software (including shadow projection on the right). For strain designation, see Table 1. (Scale bars, 50 µm).
(TIF)

Table S1 **Oligonucleotides used in this study.**
(DOCX)
