# Supplementary figures and images for: Pilus Biogenesis in Lactococcus lactis: Molecular Characterization and Role in Aggregation and Biofilm Formation
Source: PLoS One. 2012 Dec 6;7(12):e50989. doi: 10.1371/journal.pone.0050989 (PMC3516528; doi:10.1371/journal.pone.0050989)

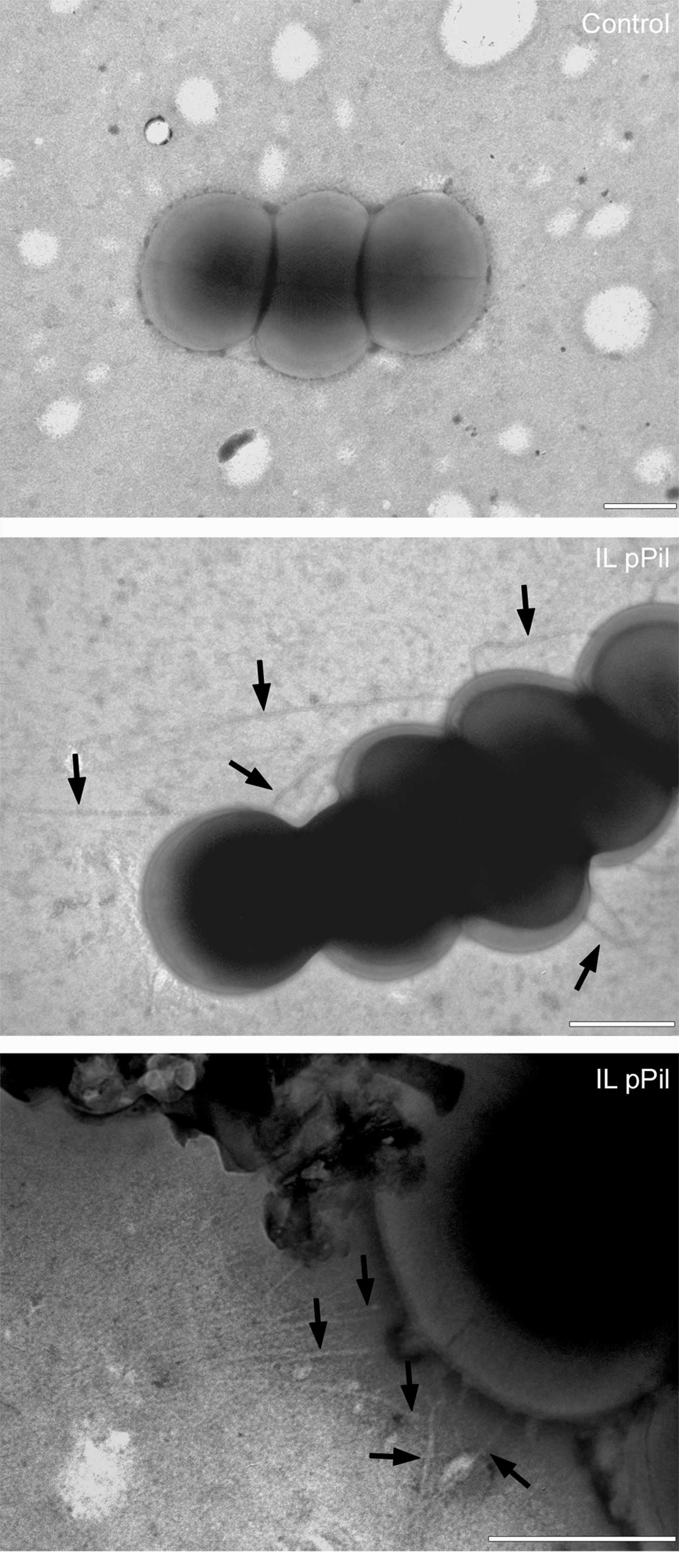

Supplement: Figure S1 — TEM images of negatively stained L. lactis strains. Control refers to L. lactis IL1403 strain harboring pIL253 plasmid and IL pPil to L. lactis IL1403 strain in which the pil operon is over-expressed. Pili are indicated by black arrows. (Scale bars, 500 nm). (TIF) [file pone.0050989.s001.tif]

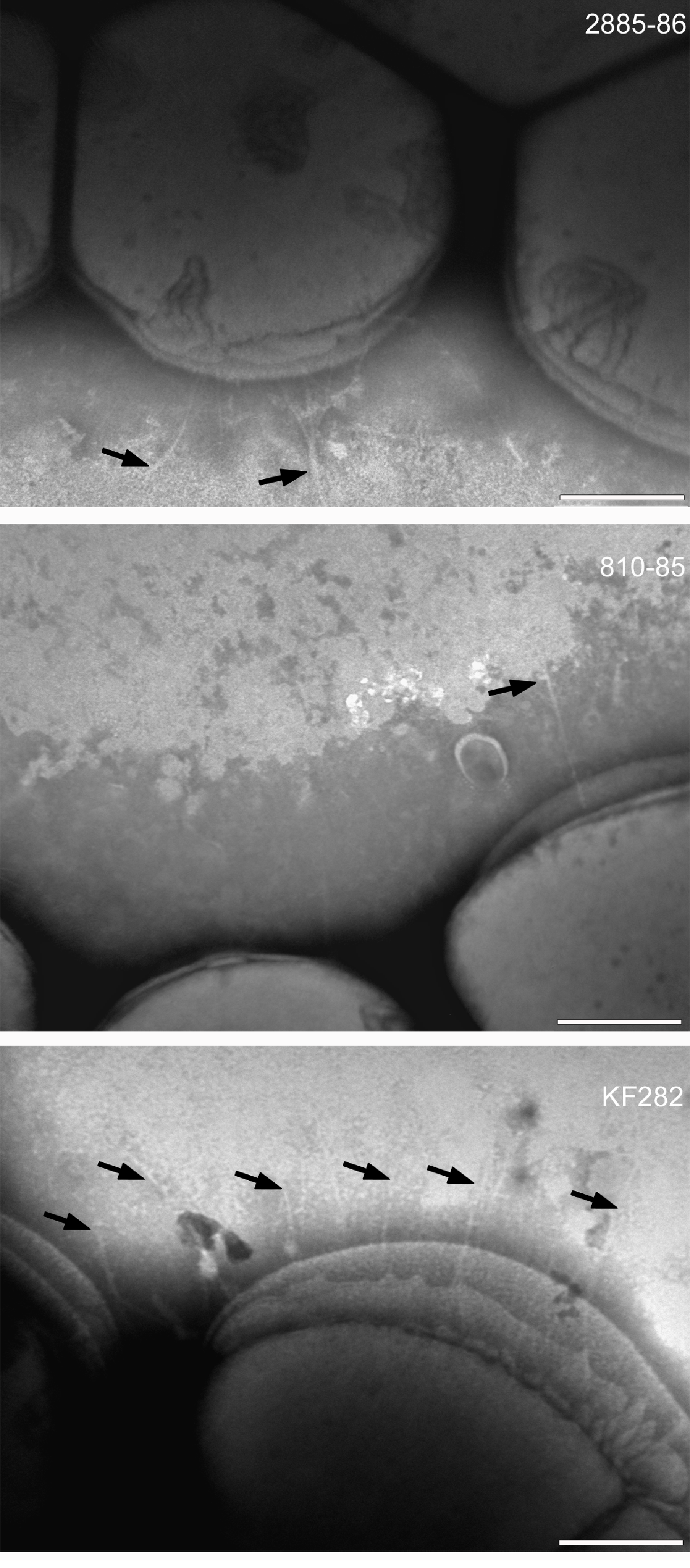

Supplement: Figure S2 — TEM images of negatively stained L. lactis strains. Pili are indicated by black arrows. Strain designation is indicated at the up and right side of the images (see Table 1). (Scale bars, 200 nm). (TIF) [file pone.0050989.s002.tif]

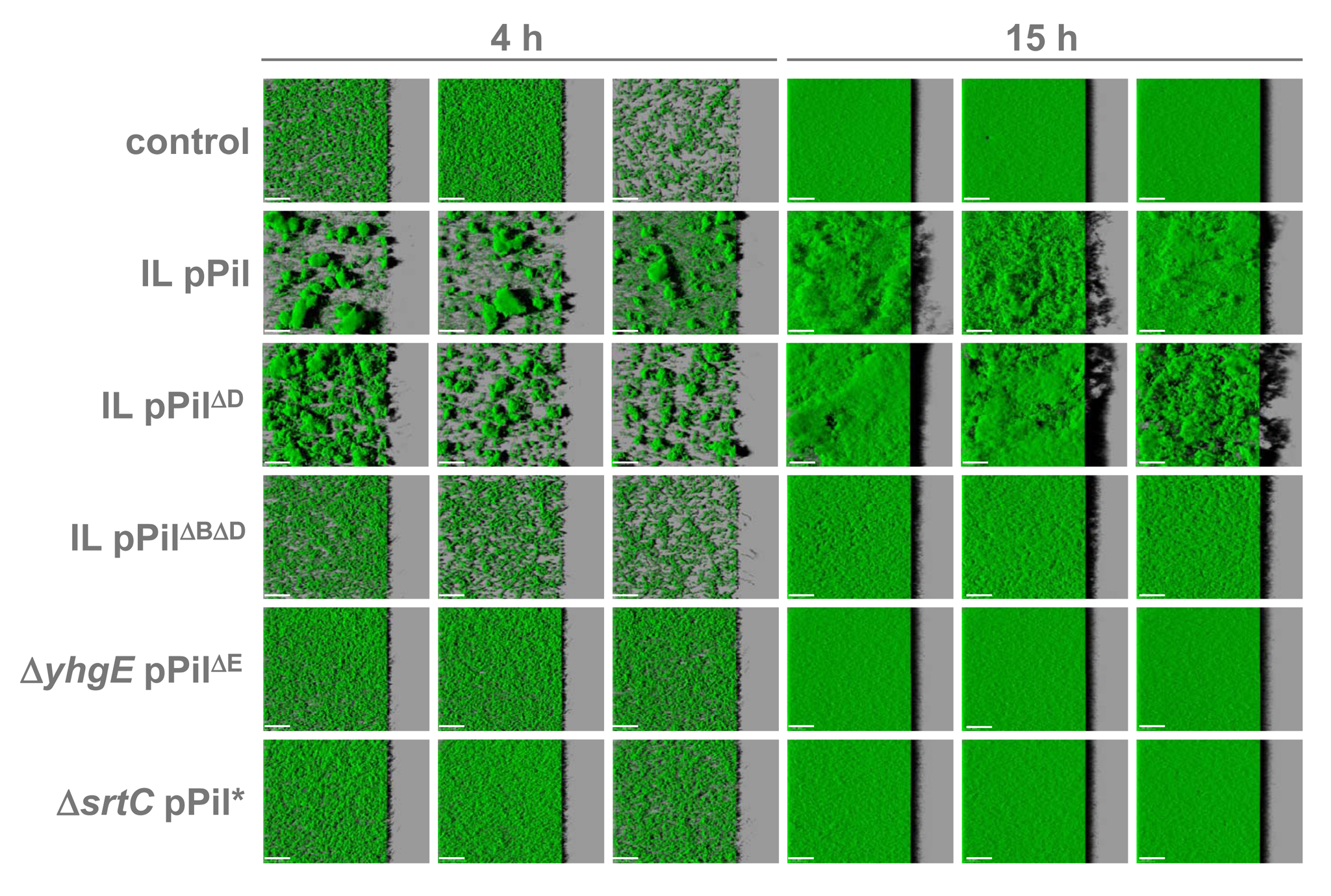

Supplement: Figure S3 — Three-dimensional biofilm structures of L. lactis strains obtained from confocal image series. Control L. lactis IL1403, IL pPil, and derivative strains were analyzed after 4 and 15 h growth in microtiter plates at 30°C under static conditions. Three images per strain are presented. Image analyses were performed using IMARIS software (including shadow projection on the right). For strain designation, see Table 1. (Scale bars, 50 µm). (TIF) [file pone.0050989.s003.tif]
