# Supplementary material for: Pilus Biogenesis in Lactococcus lactis: Molecular Characterization and Role in Aggregation and Biofilm Formation
Source: PLoS One. 2012 Dec 6;7(12):e50989. doi: 10.1371/journal.pone.0050989 (PMC3516528; doi:10.1371/journal.pone.0050989)
Supplement: Table S1 — Oligonucleotides used in this study. (DOCX) [file pone.0050989.s004.docx]

**Table S1. Oligonucleotides used in this study**

| **Designation** | **Sequences*** |
| --- | --- |
| yhhB2 | TGAATCATCCTTACTTTTAGGAGCTT |
| PrSorB4 | GCTACACCAGTTTTTAGTAATAATCCAAA |
| yhgC1 | GAGTTCAATTTTGGGGATTTTTCGAG |
| yhgD2 | CCTGTCCCGCCAACAAAACC |
| PrSorA2 | GATCAAT**GGCC**AGCTGTTCTTTCACCAGTAACTAGTA (***Not*I)** |
| PrSorD3 | GTCCCGTGACCAACACTCAA |
| yhgD6 | CCAAGTAGTGAAATTACATCAAGTACC |
| yhhB3 | GTTTCCTAAATTATCCAAGGGGAG |
| SrtB1 | CAGT**CTCGAG**CTGTAGCTCCAGTCACTACA (***Xho*I**) |
| SrtB2 | GCTG**ATCGAT**GCCATCATCCCCTCCTAAT (***Cla*I**) |
| SrtB3 | GCTGCG**ACTAGT**GGAGGGATAATTGACAAAAAGTA (***Spe*I**) |
| SrtB4 | GTAT**CCGCGG**TTACTGACCCAGTTGCTCAA (***Sac*II**) |
| yhgD3 | GG**GGTACC**GGCACCTAACACTGTAAATGTAAC (***Kpn*I**) |
| yhgDE | CCG**CTCGAG**TTAAGTTTCATTTGTTTATCTCCATTTTTATTTGATT (***Xho*I**) |
| yhgE8 | TCCC**CCCGGG**TAACGAAGAAGAAGTTGTGAAATAAAAAACTTT (***Xma*I**) |
| yhhB4 | TCC**CCGCGG**TGTAGTCCATCATTTGTCGTCTGA (***Sac*II**) |
| yhgD9 | AT**GGGCCC**CATGTATCGTTCTAAGCATCAAAAAGGT (***Apa*I**) |
| yhhC3 | AGGAACTGGACATGCTGTCGG |
| yhgD1 | CTCGACACACTCGTCCTTACG |
| yhgDE1 | TTCCATTAAAGATAAAGTTTTTTGTTTATCTCCATTTTTATTTGATTGAGTTGTG (yhhA9) |
| yhhA9 | AATAAAAATGGAGATAAACAAAAAACTTTATCTTTAATGGAAGTGGTAAACTAG (yhgDE1) |
| yhhA10 | GAGGCACATGTCCAACCCCTA |
| yhhA6 | TAGGGGTTGGACATGTGCCTCAAT |
| yhhA7 | ATTAATACCTGTTGGATAAGCAGTGACTAAAGTCACCAAATCTCGTC (yhhA8) |
| yhhA8 | TGGTGACTTTAGTCACTGCTTATCCAACAGGTATTAATAACAAAAGATTACTAG (yhhA7) |
| pVE5618a | TCGACCTCGAGCGGCCG |
| yhgE9 | AT**GGGCCC**ATGAAACTTAACTCACTTAATAAAAACTTTGCTTTG (***Apa*I**) |
| yhgD8 | CACCATGCCTAAAACAGAGAAAGCGCAAG |
| yhgD13 | CGCTGCCGTTGTAGCACC |
| yhgE1 | CACCATGACAACTAATGTAGCGGACCAAG |
| yhgE2 | TGGAGCGGCAACTTCGACA |
| yhhB1 | CACCATGGTAAAATATGGGCTAAGCCCTG |

***,** restriction sites are in bold, corresponding enzymes are indicated in parenthesis, sequences that are complementary to the sequence of the corresponding oligonucleotide indicated in parenthesis are underlined, and nucleotides corresponding to the Cys/Ala substitution in SrtC are shaded in grey.
